# Wrist Rigidity Evaluation in Parkinson’s Disease: A Scoping Review

**DOI:** 10.3390/healthcare10112178

**Published:** 2022-10-31

**Authors:** Camille Marques Alves, Andressa Rastrelo Rezende, Isabela Alves Marques, Luanne Cardoso Mendes, Angela Abreu Rosa de Sá, Marcus Fraga Vieira, Edgard Afonso Lamounier Júnior, Adriano Alves Pereira, Fábio Henrique Monteiro Oliveira, Luciane Pascucci Sande de Souza, Guy Bourhis, Pierre Pino, Adriano de Oliveira Andrade, Yann Morère, Eduardo Lázaro Martins Naves

**Affiliations:** 1 Assistive Technology Laboratory (NTA), Faculty of Electrical Engineering, Federal University of Uberlândia, Uberlândia 38400-902, Brazil; 2 Laboratoire de Conception, d’Optimisation et de Modélisation des Systèmes (LCOMS), Université de Lorraine, 57070 Metz, France; 3 Centre for Innovation and Technology Assessment in Health (NIATS), Faculty of Electrical Engineering, Federal University of Uberlândia, Uberlândia 38400-902, Brazil; 4 Bioengineering and Biomechanics Laboratory (Labioeng), Federal University of Goiás, Goiânia 74690-900, Brazil; 5 Computer Graphics Laboratory (CG), Faculty of Electrical Engineering, Federal University of Uberlândia, Uberlândia 38400-902, Brazil; 6 Federal Institute of Education, Science and Technology of Brasília—Brasília Campus (IFB), Brasília 70830-450, Brazil; 7 Applied Physical Therapy Department, Federal University of Triangulo Mineiro, Uberaba 38025-180, Brazil

**Keywords:** rigidity assessment, Parkinson’s disease, wrist rigidity

## Abstract

(1) Background: One of the main cardinal signs of Parkinson’s disease (PD) is rigidity, whose assessment is important for monitoring the patient’s recovery. The wrist is one of the joints most affected by this symptom, which has a great impact on activities of daily living and consequently on quality of life. The assessment of rigidity is traditionally made by clinical scales, which have limitations due to their subjectivity and low intra- and inter-examiner reliability. (2) Objectives: To compile the main methods used to assess wrist rigidity in PD and to study their validity and reliability, a scope review was conducted. (3) Methods: PubMed, IEEE/IET Electronic Library, Web of Science, Scopus, Cochrane, Bireme, Google Scholar and Science Direct databases were used. (4) Results: Twenty-eight studies were included. The studies presented several methods for quantitative assessment of rigidity using instruments such as force and inertial sensors. (5) Conclusions: Such methods present good correlation with clinical scales and are useful for detecting and monitoring rigidity. However, the development of a standard quantitative method for assessing rigidity in clinical practice remains a challenge.

## 1. Introduction

Parkinson disease (PD) is one of the most common neurodegenerative disorders, affecting 2–3% of the population above 65 years old [1]. PD is characterized by the presence of Lewy bodies containing α-synuclein in dopaminergic neurons in the substantia nigra of the midbrain [2]. The resulting loss of dopaminergic neurons impairs voluntary and involuntary movement, as well as autonomic [3]. This accumulation of α-synuclein becomes more widespread in the brain during PD progression [4]. Motor disorders, slow initiation of voluntary movements, and increasing reduction in the speed and amplitude of repetitive activities (bradykinesia) are clinical signs of PD. Rigidity, resting tremor, or postural instability are also common signs present in PD, whose evaluation is required for diagnosis [5].

Rigidity is a cardinal sign of PD, affecting up to 89% of the patients [6]. As a clinical sign, rigidity refers to an increased resistance to passive stretching of a muscle and is considered constant throughout the range tested [7]. The main signs of rigidity include both monosynaptic and long-latency stretch reflex exacerbation [8]. One of the distinguishing neurophysiological features of patients with Parkinson’s disease is an increase in long-latency stretch reflexes [9]. The intrinsic component of rigidity, on the other hand, includes the viscoelastic properties of muscle fibers and passive connective tissues. In this sense, evidence suggests that both the neural reflex and intrinsic mechanisms work in conjunction to cause parkinsonian rigidity. Differentiating and quantifying the two components contributes to a better understanding of the components’ underlying rigidity.

Wrist rigidity can be observed during passive flexion-extension movement as a resistance to movement [1]. The wrist is one of the most used joints when performing basic tasks, such as handling objects or feeding and is an essential component in performing the activities of daily living (ADLs) associated with a high quality of life [10,11]. In this sense, the assessment of wrist rigidity, performed through an efficient approach, is of great interest, to quantify the degree of limb impairment, as well as to evaluate the efficacy of a particular treatment, in addition to personalize the rehabilitation process [12]. Wrist rigidity is a clinically significant feature that affects primarily flexion and extension movements [13], besides visibly affecting wrist rotations and influencing forearm pronation and supination [14].

The clinical evaluation of PD motor signs, particularly rigidity, is based on a scoring system that employs clinical scales, such as the Unified Parkinson’s Disease Rating Scale (UPDRS) [15], which are subject to inaccuracies as they rely on the therapist’s experience [16,17]. To assess rigidity, the clinician should passively rotate the patient’s wrist and observe if there is resistance to movement. Based on his interpretation of the feeling of resistance to movement, the therapist defines the patient’s rigidity level in a range from 0 (normal) to 4 (severe) [18]. Another disadvantage of this type of scale is the high variability of intra- and inter-evaluator results. This reduces the objectivity of clinical rating scales [19]. Recent research has shown that efforts are being made to establish objective methods for assessing muscle tone and rigidity, which are essential for obtaining quantifiable, reliable and reproducible data [20].

The most used clinical scales, such as the UPDRS, can be viewed as a qualitative assessment method [21]. The main reason for using these scales is their simplicity of application, which makes them popular and easy to use in clinical practice. However, this same attribute is the most criticized, as it depends entirely on precise clinical observations [6,19]. In this sense, the great challenge is to develop quantitative methods that are easy to interpret and implement in clinical practice, and independent of the therapist’s experience.

Despite significant progress, assessing rigidity in people with PD remains difficult, and just a few studies have focused on quantitative assessment devices for the wrist joint evaluation [6]. Considering the importance of improving the methods for assessing wrist rigidity in people with Parkinson’s disease, it is necessary to review relevant published articles that have already studied this subject.

Some reviews have been made before [6,22,23,24,25], however, no direct solutions for wrist rigidity were presented, as the studies focused on other symptoms of Parkinson’s disease, such as bradykinesia and tremor. Additionally, they concentrated on other joints. In addition, each article emphasizes the significance of establishing a quantitative measurement standard for these signals using sensors and innovative technologies. In this regard, the purpose of this research is to identify all current methods for assessing wrist rigidity and their state of the development.

## 2. Materials and Methods

### 2.1. Protocol and Registration

This review was registered in OSF (DOI 10.17605/OSF.IO/4AGQ6). A scoping review was performed on studies that evaluated rigidity, following the methodological recommendations of the Joanna Briggs Institute for systematic scoping reviews [26]. The target patient population was individuals with PD and the concept of interest was the assessment of wrist rigidity. Studies were included in the review if they met the following criteria:

1. Studies that addressed the evaluation of the wrist rigidity in people with Parkinson’s disease.
2. Participants with Parkinson’s disease of both sex and any age.
3. Randomized clinical trials, or randomized controlled trials, or clinical trial, or case reports, or cohort study.
4. Full text written in English.

Exclusion criteria were:

1. Incomplete studies, that do not present results.
2. Studies that address the assessment of wrist rigidity in people with Parkinson’s disease, but not the main topic.

### 2.2. Search Strategy

The search strategy followed the Preferred Reporting Items for Systematic review and Meta-Analysis (PRISMA) [27] guidelines. An electronic search was conducted in PubMed, IEEE/IET Electronic Library, Web of Science, Scopus, Cochrane, Bireme, Google Scholar and Science Direct databases.

Key terms were searched within the paper title, abstract and keywords, using conjunctions “OR” and “AND”. The keywords and query utilized in all databases were: Parkinson AND (Evaluation OR Assessment OR Exoskeleton) AND (Stiffness OR Rigidity) AND (Wrist OR Hand). There was no year limitation up to July of 2022.

### 2.3. Study Selection

After applying the search strategy in all databases, the studies were saved and organized in Rayyan—Intelligent Systematic Review [28]. The selection process of the studies was performed following the steps below:

1. First, the title and abstract of the articles were read and analysed considering the inclusion criteria. This analysis was performed by two investigators.
2. Next, the selected studies were read in full, performing a second filtering, and those that did not fit the inclusion criteria were excluded. This selection was performed by two researchers.
3. Finally, data and characteristics were extracted, in addition to the quality evaluation of the selected studies.

**Figure 1:**
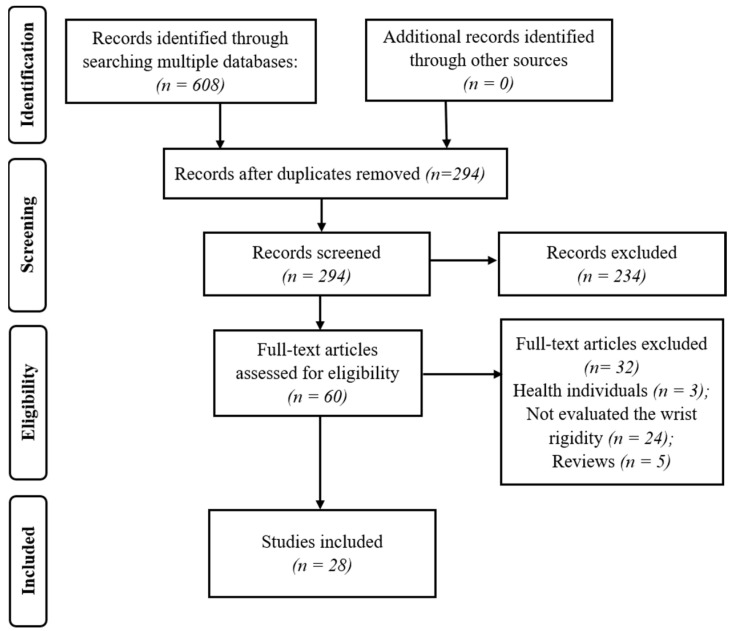
PRISMA flow diagram illustrating the studies selection.

The results are presented in Figure 1. Eliminating the duplicates, we have found 294 studies from 8 databases. From those, screening the studies by title and abstract, we evaluated whether the study met the inclusion criteria, that is, in addition to citing, it measured wrist rigidity in individuals with PD. Since, several articles cited wrist rigidity but the focus was to measure in another joint, as elbow [29]. Thus, we excluded 234 studies. In the third step, the full-text screening of the 59 studies was performed, and 28 studies were included for analysis. The exclusion of the 32 studies occurred for population criteria (3), outcomes (24), and study design (5) (Figure 1).

### 2.4. Data Analysis

Two authors (Alves C.M. and Rezende A.R.) extracted data from the selected articles. Disagreements between reviewers were resolved by consensus. If no consensus could be reached, a third reviewer (Marques I.A.) would decide. The information from the selected studies was organized in tables by method of evaluation, with each reference corresponding to a study. The information collected were: type of study, year of publication, sample size and characteristics and type of evaluation method.

## 3. Results

### 3.1. Description of the Included Studies

**Figure 2:**
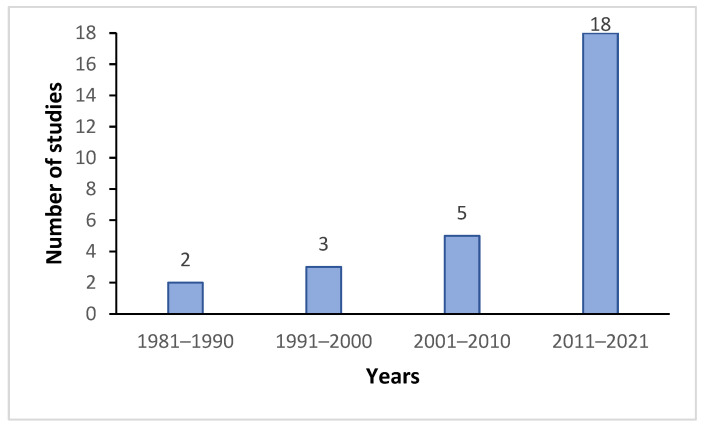
Number of selected studies per decade.

The selected studies include 15 case series, 4 cross-sectional studies, 7 case–control and 2 cohort studies. The year of publication of the selected studies ranged from 1988 to 2021. Figure 2 depicts the number of studies published in each decade. There is an increase in wrist rigidity in Parkinson’s disease research, particularly in the past decade, when 18 out of the 28 selected studies were published.

**Figure 3:**
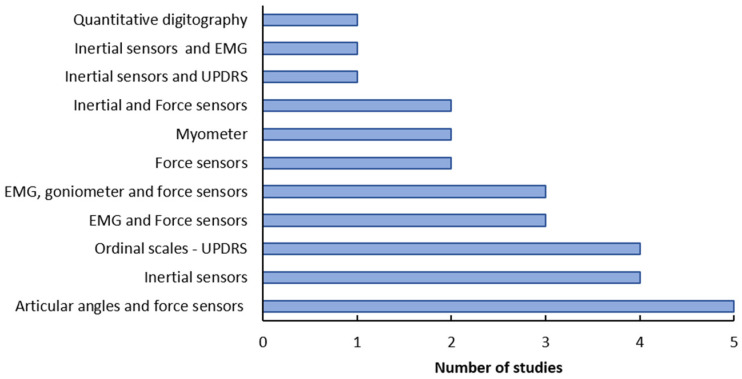
Methods used to assess wrist rigidity in the selected studies.

In the selected studies, several kinds of sensors were used to assess wrist rigidity in patients with PD (Figure 3). Indeed, some studies used a combination of sensors to propose a method with greater reliability, but easy to use. The most common combination of sensors was goniometers (articular angles) and force sensors, as observed in five of the selected studies [13,30,31,32,33]. An important point is that the results of the articles reviewed cannot be directly compared with each other, since there is great variability in the methods used by each group.

#### 3.1.1. Methods Evaluating Muscular Signals

Eight studies were included, which are summarized in Table 1. In three different studies, EMG was used in conjunction with goniometers (articular angles) and force sensors [34,35,36], two [31] used with force sensor only and one [37] with inertial sensors.

Although not all studies present correlation results with the clinical scales, electromyography (EMG) has been widely used to study the muscle physiology of patients with PD, showing good correlation with clinical scales that assess rigidity. Some studies have found a highly significant correlation between normalized stretch-related EMG and the degree of rigidity. Viscous damping constant had the best correlation (=0.77 ± 0.22) with clinical measures and the greatest number of limbs with significant positive correlation (57%) [35]. Clinical and device assessments that used EMG were significantly correlated, although the relationship was not strong (Spearman’s rho = 0.382, *p* = 0.002) [36]. 

Two studies used the Myotonometer, a device that measures the biomechanical and viscoelastic properties of muscle [38,39]. The stretches were done manually by the examiner in both studies. Natural oscillation data are obtained using this method, which measures acceleration and “voltage” parameters. This technique estimates muscle tone (Hz) and rigidity (N/m) [40]. Both studies did not present significant positive correlations between clinical rigidity scores and viscoelastic stiffness values (before PWM-s: ρ = 0.12; *p* = 0.3; and after PWM-s: ρ = 0.284; *p* = 0.1).

#### 3.1.2. Methods Evaluating Capture of Movement 

Eight studies were included, which are summarized in Table 2. Inertial sensors were used in four studies [41,42,43,44], in combination with EMG in one study [37], and combined with force sensors in two other studies [15,45]. The study [46] utilized the UPDRS. Three studies applied the evaluation during the DBS surgery [41,43,44], and applied a classification model to differentiate rigid and non-rigid states.

Despite the vast majority of studies using IMU sensor not showing correlation values with clinical scales, some devices were able to correctly classify 83.9% of the evaluated signals, compared to UPDRS [41]. Mann–Whitney rank order comparisons revealed that the median rigidity score for clinically classified rigid patients was significantly higher than the median control (U = 17, *p* < 0.0001) or the median rigidity score of the non-rigid patient (U = 15, *p* = 0.0001) [32].

#### 3.1.3. Methods That Used Mechanized and Force Sensors

Seven studies were included, which are summarized in Table 3. In studies of Xia et al. [13,31] the stretching velocity was controlled at 50°/s. Teräväinen et al. [33] proposed a comparison between different velocities and found that higher velocities were the most sensitive for detecting parkinsonian rigidity. Force sensors alone were in two studies [20,47], and combined with electromyography (EMG) analysis in three studies [16,48,49]. Powell et al. and Xia et al. [16,49] also performed in different velocities and found greater rigidity at higher speeds.

Most of the studies did not show comparison results with the clinical scales. However, it was observed that the *p* value obtained in the evaluation of the differences between PD OFF and PD ON using a device (*p* = 0.016) is lower than that obtained by the MDSUPDRS Part III score (*p* = 0.031). This may lead to the hypothesis of a greater sensitivity of the equipment in relation to the clinical score [30]. Spearman’s correlation analysis showed mild to moderate correlations between device scores and UPDRS rigidity scores in the off-medication state (q = 0.482, *p* = 0.111 for the slowest movement; q = 0.578, *p* = 0.049 for the faster movement). In Medication Status, there was a low correlation between device and clinical degrees of rigidity for slower movement (q = 0.324, *p* = 0.305) and no correlation was found for rapid movement (q = 0.248, *p* = 0.437) [20].

#### 3.1.4. Ordinal Scales

Five studies were included, which are summarized in Table 4. Despite the vast range of sensors utilized, five studies [50,51,52,53] employed ordinal scales to assess individuals. Although these scales are widely used in clinical settings, they have several drawbacks, such as relying on the therapist’s experience, resulting in subjective and unreliable assessment. 

One study applied a new technique using quantitative digitography [54], in which the person performed movements on a keyboard and the application force was measured, at the end they were able to compare rigidity and tremor parameters with the UPDRS. There were significant associations between the rigidity sub-score and release slope (ρ = −0.43, *p* = 9.78 × 10^−9^).

### 3.2. Applicability of the Methods in Clinical Practice

An extremely important factor in the application of new rigidity evaluation methods is their suitability in clinical practice and their applicability in different clinical scenarios. In addition, it is necessary to analyse the methods that can be applied at home, since, even in times of a pandemic like COVID-19, this type of evaluation is important. In this sense, methods that can be adapted to be applied at home are more interesting.

**Figure 4:**
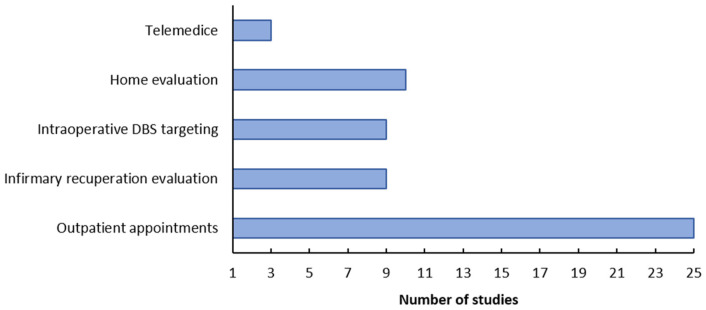
Applicability of the methods.

Analysing the articles found in this review, the applicability of the methods in different clinical scenarios was evaluated, such as: outpatient appointments, infirmary recuperation evaluation, intraoperative DBS targeting, telemedicine and home evaluation. Figure 4 shows the applicability of the methods found, in different scenarios. It can be noted that there are still few devices that can be implemented in telemedicine, while the vast majority can be used in outpatient appointments.

## 4. Discussion

This scoping review included 28 studies investigating the most used methods for wrist rigidity evaluation in individuals with Parkinson’s disease. The most studies presented quantitative methods associated with UPDRS, which is a scale commonly used for this assessment in clinical practice [55]. It can also be noticed a greater concentration of the selected studies in the past 10 years, which denotes an increasing demand for objective methods that present more reliable results [56]. One possible explanation for this increase in publications is the need for therapists to seek out and develop alternative methods that are intuitive and easy to interpret [56]. Furthermore, they must present data that allows for a more accurate tracking of the patient’s progress.

Due to the subjective nature of clinical assessments and the small group of patients, it would be difficult to expect a direct correlation between UPDRS values and devices that measure rigidity. The low sensitivity and high variability of the clinical assessment may also explain the relatively modest correlation between the mechanical and clinical assessments of rigidity. Due to differences in the design of measuring devices, the studies are not directly comparable, however, the methods appear to allow researchers to objectively describe changes in stiffness in patients with PD. Most studies presented instruments with the possibility of obtaining objective results for detecting and quantifying rigidity compared to healthy individuals. In this sense, such instruments can be considered as useful tools in the assessment of wrist rigidity in people with PD.

The neural and non-neural components in determining rigidity are still being debated, even though both contribute to the unique nature of Parkinsonian rigidity. Non-neural components correspond to the mechanical properties of muscle fibers, whereas neural components, such as shortening reflexes, are inherent in pathophysiology [13,31,49]. These later aspects can be detected by EMG and force sensors [16,48,49]. Studies that used a combination of such sensors exhibited better results, with a good correlation with scales used in clinical practice. Furthermore, EMG and force measures are more representative of the neurological source of the motor symptoms [57].

On the other hand, other studies used inertial sensors and goniometers (joint angles) to assess wrist rigidity [41,42,43,44]. Inertial sensors are currently used for angular velocity measurement in rigidity assessment in PD [58]. The results in the cited studies indicate that the viscosity and elasticity of the muscle in the rigidity condition are higher than in the healthy condition (relaxed state) [42]. In this sense, the combination of data from inertial sensors and joint angles can detect the on-off fluctuations of parkinsonian rigidity. Range of motion is an indirect measure of the progression of rigidity in Parkinson’s disease. In most cases, rigidity is accompanied by a decrease in joint range of motion [59].

Regarding the protocols used in the studies, some used different stretching speeds for the rigidity evaluation [16,33,37,49,52]. Some approaches suggest that parkinsonian rigidity is considered velocity-independent, in contrast to spasticity, which is velocity-dependent [52,60]. However, more recent studies have raised questions about the accuracy of this point of view [6,16]. These studies concluded that rigidity depends on the angular velocity and articular amplitude of the applied mobilization and Parkinsonian rigidity is modulated by the amplitude and rate of muscle stretch. The differences between the experimental protocols leads us to different results, while some studies such Powell et al. and Xia et al. [16,49], applied and analysed different stretching speeds, others such as Meara et al. [52] applied different speeds but did not compare the results. These findings may help to understand the biomechanical foundations and physiological characteristics of rigidity and may assist in the development of new methods to assess clinical rigidity in Parkinson’s disease.

The way in which passive extension/flexion is performed is a critical aspect of objective rigidity assessment. Servomotor manipulation was used in some studies [13,20,30,48,49], while manual manipulation was used in others [15,38,45,51]. When done manually, it is difficult to ensure that the therapist is stretching at the appropriate speed. As a result, studying the relationship between speed and rigidity is difficult.

A parameter of great relevance in this research is the incorporation of these devices in clinical practice, and its usability in different clinical scenarios. Although most of the devices found have not been applied in clinical practice, it is mentioned where they could be used. According to the analyses carried out, regarding the applicability of these methods, most devices can be applied in outpatient appointments, and few in the other scenarios. One of the reasons for this is the size of the devices presented, which often makes it difficult to carry and mount them in other environments, outside the clinical offices. In addition, to have a better view of these factors, it is necessary to perform some usability tests, in addition, the complexity of the proposed devices must be evaluated [61].

Another relevant parameter is the suitability of these devices for use in telemedicine, that is, for use by patients at home without the physical presence of therapists. Recent studies discuss the limitation of measuring rigidity through remote systems, since it is a parameter that must be evaluated personally, through physical contact with the patient. In this review we found three papers [20,43,54] that discuss this usability, and after some validation tests, the technology proposed by them could be implemented remotely as long as the patient was trained with the device.

Some knowledge gaps were found in this review. Comparative studies were not performed analysing the different instruments compiled in this review. It would be interesting to analyse the diagnostic accuracy to decide which of these methods is the gold standard for identifying stiffness in PD. Despite a recent increase in the number of studies developing new technologies for the quantitative assessment of rigidity, it has not yet been discussed how to incorporate them into clinical practice. More tests should be performed in clinical practice, to understand how these new devices would be incorporated during clinical evaluation. Another important aspect is the evaluation protocol, most articles apply passive flexion and extension of the wrist, but there is no consensus on velocity applied. It is also necessary to determine whether assessments should be performed in the “on” or “off” period of medication.

### Main Limitations and Clinical Implications

The present study has some limitations. The quality of the studies was not examined as these factors are not included in a scoping review. This should be verified in future studies. Another limitation was that the review contained only studies that evaluated wrist rigidity, not considering other joints. This choice was made since there are few review articles focused on this articulation. Furthermore, the wrist is an essential component in performing the activities of daily living (ADLs) associated with a high quality of life. Furthermore, this study can help in the development of new technologies focused on the evaluation of wrist rigidity. Another limitation was the lack of quantitative data comparing the proposed methods with the clinical scales, which made it impossible to carry out a more complete statistical analysis of the methods.

Rigidity is one of the most common symptoms of Parkinson’s disease, and the wrist joint is frequently affected. It is a joint that directly affects the ability to perform everyday tasks and the quality of life of people with the disease. The diagnosis and follow-up of patients rely heavily on the assessment of rigidity in clinical practice. When this evaluation is done quantitatively, it is possible to track patient treatment more accurately and test new medicines with greater safety. However, the primary concern is how to implement these new technologies into clinical practice. Thus, additional tests with existing technologies are required, with an emphasis on applications that are simple to use. Additionally, the results must be reliable and simple to interpret.

## 5. Conclusions

The main findings of this study showed that there is an increase in the number of studies focused on objective methods for assessing wrist rigidity in Parkinson’s disease in the last few years. It was possible to observe an increasing search for methods that provide reliable results and that consider all aspects of rigidity. Several types of medical devices have been proposed, but none has been clinically validated or subjected to an industrial validation procedure. Therefore, additional analysis using existing methods is required.

It can be observed that none of the proposed equipment was incorporated into clinical practice. Probably due to the ease of applying clinical scales, and also the lack of more precise studies with these new technologies. It is necessary to apply the proposed methods in a larger number of individuals and carry out tests during procedures performed in the clinic. Another aspect that should be better determined is the protocol to be applied in this evaluation.

## Figures and Tables

**Table 1:** Characteristics of studies using EMG.

Study	Year	Study Type	Participants	Method of Evaluation	Evaluation Protocol	Results
[38]	2017	Case series	15 patients in an advanced stage of PD	Myotonometer (muscle biomechanical and viscoelastic properties)	During the “off” state, one examiner flexed and extended the wrist joint for 10 times and the measurements were taken for one second.	Effective deep brain stimulation and increased rigidity can significantly change viscoelastic rigidity in the resting muscles in patients with PD.
[34]	2016	Case series	18 PD patients	EMG, articular angle and force sensor	Participants were tested in the “on” and “off” states. Rigidity was quantified by rigidity work score and slopes of the moment-angle plots during flexion and extension.	The presentation of rigidity used in the determination of diagnosis, treatment and prognosis in PD will not be affected by the continuous nature of the movement trajectory used during clinical assessment.
[35]	2014	Case series	8 PD patients	EMG, articular angle and force sensor	Rigidity was evaluated during DBS surgery. The therapist performed six cycles of flexion and extension movement imposed on the wrist.	The system is capable of quantifying intra-operative stiffness and is useful for adjusting the position of the electrodes and the stimulation parameters.
[36]	2012	Case series	12 PD patients	EMG, articular angle and force sensor	The rigidity was evaluated by flexion and extension passively of the wrist. A maximum of 14 cycles per condition was performed.	The study provides a link between oscillatory activity at low frequency and PD stiffness, and validates a method for quantifying stiffness in the wrist.
[16]	2012	Case series	18 PD patients	EMG and force sensor	Subjects’ tested hand was passively displaced. Rigidity was quantified by normalized work scores and angular impulses for flexion and extension.	The larger displacement amplitude and the higher velocity were associated with significantly greater rigidity, increased EMG ratio and mean EMG of stretched muscles.
[39]	2011	Case series	6 male PD patients	Myotonometer	The study was carried out in “off” state. Rigidity was measured through Myotonometer. The examiner flexed and extend the joint 10 times.	The study supports the use of myotonometry for objective quantification of rigidity. The tool may be helpful for optimizing DBS settings in PD.
[31]	2004	Case series	6 PD patients	EMG and force sensor	The motor moved the wrist joint in movement of flexion and extension. Patients were evaluated in “on” and “off” state.	The shortening reaction was reduced after the medication.
[37]	1992	Cross-sectional	9 PD patients and 10 healthy subjects	EMG and inertial sensors	EMG was recorded during the flexion and extension movements of the wrist joint. The patients were evaluated in “on” and “off” states.	EMG indicated that mild and moderate rigidity was consistently associated with increased stretch-related activity compared with non-rigid conditions.

**Table 2:** Characteristics of Studies that used Capture of Movement.

Study	Year	Study Type	Participants	Method of Evaluation	Evaluation Protocol	Results
[42]	2021	Case control	15 individuals with PD and 12 healthy	Inertial sensors	Inertial sensors were used on the hand and forearm. Different features were used to compare the values of sensitivity, specificity, precision, and accuracy of the classifiers.	The best performance for sensitivity and accuracy (0.875 and 0.800, respectively) was found in the SVM classifier, and for specificity and precision (0.933 and 0.917) was associated with the RF.
[46]	2019	Case series	46 community-dwelling people with PD	Inertial sensors and UPDRS	The participants were instructed to wear ActiGraphs for 7 consecutive days, for 24 h a day.	Wrist monitors may overestimate activity; however, activity monitors can be used if the aim is to monitor change rather than accurately record activity.
[43]	2019	Cohort study	22 bilateral DBS surgeries of patients with PD	Inertial sensors	The system was used during the DBS surgery. The rigidity was assessed by imposition of passive wrist flexion.	The polynomial models were tested for a larger number of patients. An accuracy of 78% and a rigidity classification error of 3.5% was achieved.
[45]	2017	Case series	4 PD patients	Inertial sensors and force sensor	The hand was passively moved by the examiner, who had a moment sensor attached to his hand. Moment arm was measured using a ruler.	The PowerGlove measured a difference in off- vs. on-medication condition in all tasks in the patients with most of its outcome parameters.
[44]	2016	Case series	17 PD patients	Inertial sensors	The system was used during the DBS surgery. The rigidity was assessed by imposition of passive wrist flexion.	The system correctly classified 82.0% of the signals (mean error of 3.4%), which supports the reliability of this solution.
[41]	2015	Cohort study	6 PD patients subjected to DBS surgery	Inertial sensors	The system was used during the DBS surgery. The rigidity was assessed by imposition of passive wrist flexion.	The descriptor distinguished between non-rigid and rigid states, and the classification model labelled correctly 83.9% of the signals.
[15]	2001	Case series	4 PD patients	Articular angles and inertial sensors	The examiner repeatedly flexes and extends the joint. A solid-state piezoelectric gyroscope monitors the angular velocity from which is computed displacement.	Examiners tended to overrate rigidity on the UPDRS. Mechanical impedance was nonlinearly related to UPDRS ratings of rigidity at wrist.
[32]	1994	Case control	25 healthy controls and 29 PD patients	Articular angles	The therapist moved the patient’s wrist in flexion and extension while the signals were collected.	The device presented 89% of specificity, 82% sensitivity and great portability.

**Table 3:** Characteristics of Studies that used Force Sensors.

Study	Year	Study Type	Participants	Method of Evaluation	Evaluation Protocol	Results
[20]	2020	Case control	7 PD subjects and 14 healthy subjects	Force sensors	PD subjects were assessed in the “on” and “off” states. The torque perturbation was applied five times, thus leading to an overall duration of the session equal to 105 s.	The device allowed to successfully estimate the rigidity, and allowed to discriminate both Healthy subjects from PD subjects, and PD subjects in “off” condition from PD subjects in “on” condition.
[47]	2019	Case series	5 PD patients	Force sensors	It was measured at the “on” and “off” states. The wrist was evaluated 3 times without and 3 times with the Froment maneuver.	A minimum-maximum limit forming study was done for rigidity values between 0–5 using both Froment rigidity values’ scores.
[30]	2018	Case control	18 healthy and 4 PD patients	Force sensor	The wrist joint was positioned in alignment with the centre of rotation of the plate. Ten repetitions of flexion followed by extension were conducted.	The device has sufficient accuracy and sensitivity to measure the interaction torque at the wrist joint and to differentiate PD rigidity from normal muscle tone.
[48]	2011	Case control	12 subjects with PD and 8 controls	Force sensor and EMG	A servomotor executed the flexion and extension of the wrist. EMG and torque were collected. The patients were evaluated in “on” and “off” states.	Subjects showed a higher resistance in “off” compared to healthy control. The medication reduced difference in torque resistance between controls and PD patients.
[49]	2009	Case series	12 PD subjects	Force sensor and EMG	The servomotor executed the movements of flexion and extension and the velocity was constant. EMG was recorded at the flexors and extensors. Patients were evaluated in “on” and “off” state.	Torque resistance scores were more strongly correlated with the EMG ratios. The highest correlation was found between the torque resistance score and EMG during the extension at high velocity in “off” state.
[13]	2006	Cross-sectional	12 PD patients and 7 controls	Force sensor	Flexion and extension movements were carried out by a servomotor at a constant velocity. Patients were evaluated in “on” and “off” states.	The rigidity was more readily elicited in extension movements. Compared with controls the scores were higher for patients in “on” state.
[33]	1989	Case control	29 PD patients and 12 healthy subjects	Torque motor and articular angles	The individual’s hand was oscillated passively. The objective rigidity score was expressed in Newton-meter-degrees.	The rigidity was more pronounced at faster movement velocities in patients with PD.

**Table 4:** Studies that Used only Ordinal Scales.

Study	Year	Study Type	Participants	Method of Evaluation	Evaluation Protocol	Results
[54]	2021	Case control	96 individuals with PD and 42 healthy	Quantitative digitography	Individuals performed repetitive alternating finger tapping on keys, and then compared the results with UPDRS.	Quantitative digitography technology and the rigidity metric provide a important supplement to video MDS-UPDRS assessments of PD in clinical trials.
[50]	2016	Cross-sectional	31 PD patients and 32 age-gender matched healthy controls	Ordinal Scale	All patients were examined in the “on” condition. The Hoehn and Yahr (H&Y) scale and UPDRS were used to assess the severity of the disease.	Electrophysiologic parameters indicated subclinical median and ulnar nerve demyelination in patient who have longer disease duration and mild tremor and rigidity scores.
[51]	2008	Case series	17 PD patients	Ordinal scale	Assessment was performed with patients in the “on” state by two therapists using a rigidity scale.	Rigidity increased from baseline. The effect of hand opening-closing on rigidity was greater than the attentional tasks.
[52]	1993	Cross-sectional	20 PD patients and 19 healthy subjects	Ordinal scale	The patients were evaluated in “on” and “off” states. Wrist extension movement of 10° was applied at velocities 100 and 200°/s.	The results suggest that rigidity of PD cannot be uniquely attributed to the increased reflex responsiveness measured by the present techniques.
[53]	1988	Case series	20 PD subjects	Ordinal scale	Two therapists executed wrist flexion and extension movements at varying speeds.	The two evaluations presented 80% of agreement and a weighted Kappa value of 0.636.

## Data Availability

The datasets generated in the current study are not publicly available due to the ethical restrictions preventing public sharing of data. A non-identified set may be requested after approval from the Review Board of the Institution. Requests for the data may be sent to the corresponding author.

## Abbreviations

PD	Parkinson’s disease
ADL	Activities of Daily Living
MDS-UPDRS	Movement Disorder Society—Unified Parkinson’s Disease Rating Scale
EMG	Electromyography

## References

[1] Poewe W., Seppi K., Tanner C.M., Halliday G.M., Brundin P., Volkmann J., Schrag A.-E., Lang A.E. (2017). Parkinson Disease. Nat. Rev. Dis. Prim..

[2] Braak H., Braak E. (2000). Pathoanatomy of Parkinson’s Disease. Proceedings of the Journal of Neurology, Supplement.

[3] Dauer W., Przedborski S. (2003). Parkinson’s Disease: Mechanisms and Models. Neuron.

[4] Tysnes O.B., Storstein A. (2017). Epidemiology of Parkinson’s Disease. J. Neural Transm..

[5] Sveinbjornsdottir S. (2016). The Clinical Symptoms of Parkinson’s Disease. J. Neurochem..

[6] Ferreira-Sánchez M.d.R., Moreno-Verdú M., Cano-de-la-Cuerda R. (2020). Quantitative Measurement of Rigidity in Parkinson’s Disease: A Systematic Review. Sensors.

[7] Xia R., Xia R. (2011). Physiological and Biomechanical Analyses of Rigidity in Parkinson’s Disease. Etiol. Pathophysiol. Park. Dis..

[8] Berardelli A., Sabra A.F., Hallett M. (1983). Physiological Mechanisms of Rigidity in Parkinson’s Disease. J. Neurol. Neurosurg. Psychiatry.

[9] Lee R.G., Tatton W.G. (1982). Long Latency Reflexes to Imposed Displacements of the Human Wrist: Dependence on Duration of Movement. Experimental Brain Res..

[10] Moser N., O’Malley M.K., Erwin A. (2020). Importance of Wrist Movement Direction in Performing Activities of Daily Living Efficiently. Proceedings of the Annual International Conference of the IEEE Engineering in Medicine and Biology Society, EMBS.

[11] Raiano L., Di Pino G., Noccaro A., Accoto D., Formica D. (2018). Design of a Wearable Mechatronic Device to Measure the Wrist Rigidity in Parkinson’s Disease Patients. Proceedings of the IEEE RAS and EMBS International Conference on Biomedical Robotics and Biomechatronics.

[12] Ward A.B. (2000). Assessment of Muscle Tone. Age Ageing.

[13] Xia R., Markopoulou K., Puumala S.E., Rymer W.Z. (2006). A Comparison of the Effects of Imposed Extension and Flexion Movements on Parkinsonian Rigidity. Clin. Neurophysiol..

[14] Formica D., Charles S.K., Zollo L., Guglielmelli E., Hogan N., Krebs H.I. (2012). The Passive Stiffness of the Wrist and Forearm. J. Neurophysiol..

[15] Patrick S.K., Denington A.A., Gauthier M.J.A., Gillard D.M., Prochazka A. (2001). Quantification of the UPDRS Rigidity Scale. IEEE Trans. Neural Syst. Rehabil. Eng..

[16] Powell D., Joseph Threlkeld A., Fang X., Muthumani A., Xia R. (2012). Amplitude- and Velocity-Dependency of Rigidity Measured at the Wrist in Parkinson’s Disease. Clin. Neurophysiol..

[17] Goetz C.G., Fahn S., Martinez-Martin P., Poewe W., Sampaio C., Stebbins G.T., Stern M.B., Tilley B.C., Dodel R., Dubois B. (2008). MDS-UPDRS. Movement Disorders..

[18] Fleuren J.F.M., Voerman G.E., Erren-Wolters C.V., Snoek G.J., Rietman J.S., Hermens H.J., Nene A.V. (2010). Stop Using the Ashworth Scale for the Assessment of Spasticity. J. Neurol. Neurosurg. Psychiatry.

[19] Raiano L., di Pino G., di Biase L., Tombini M., Tagliamonte N.L., Formica D. (2020). PDMeter: A Wrist Wearable Device for an at-Home Assessment of the Parkinson’s Disease Rigidity. IEEE Trans. Neural Syst. Rehabil. Eng..

[20] Gottipati G., Berges A.C., Yang S., Chen C., Karlsson M.O., Plan E.L. (2019). Item Response Model Adaptation for Analyzing Data from Different Versions of Parkinson’s Disease Rating Scales. Pharm. Res..

[21] Teshuva I., Hillel I., Gazit E., Giladi N., Mirelman A., Hausdorff J.M. (2019). Using Wearables to Assess Bradykinesia and Rigidity in Patients with Parkinson’s Disease: A Focused, Narrative Review of the Literature. J. Neural Transm..

[22] Opara J.A., Małecki A., Małecka E., Socha T. (2017). Motor Assessment in Parkinson’s Disease. Ann. Agric. Environ. Med..

[23] Merola A., Sturchio A., Hacker S., Serna S., Vizcarra J.A., Marsili L., Fasano A., Espay A.J. (2018). Technology-Based Assessment of Motor and Nonmotor Phenomena in Parkinson Disease. Expert. Rev. Neurother..

[24] Adams J.L., Lizarraga K.J., Waddell E.M., Myers T.L., Jensen-Roberts S., Modica J.S., Schneider R.B. (2021). Digital Technology in Movement Disorders: Updates, Applications, and Challenges. Curr. Neurol. Neurosci. Rep..

[25] Peters M.D.J., Godfrey C.M., Khalil H., McInerney P., Parker D., Soares C.B. (2015). Guidance for Conducting Systematic Scoping Reviews. Int. J. Evid Based Health.

[26] Page M.J., McKenzie J.E., Bossuyt P.M., Boutron I., Hoffmann T.C., Mulrow C.D., Shamseer L., Tetzlaff J.M., Akl E.A., Brennan S.E. (2021). The PRISMA 2020 Statement: An Updated Guideline for Reporting Systematic Reviews. BMJ.

[27] Ouzzani M., Hammady H., Fedorowicz Z., Elmagarmid A. (2016). Rayyan—A Web and Mobile App for Systematic Reviews. Syst. Rev..

[28] Dai H., Otten B., Mehrkens J.H., D’Angelo L.T. A Portable System for Quantitative Assessment of Parkinsonian Rigidity. Proceedings of the 2013 35th Annual International Conference of the IEEE Engineering in Medicine and Biology Society (EMBC).

[29] Zito G.A., Gerber S.M., Urwyler P., Shamsollahi M.J., Pal N., Benninger D., Nef T. (2018). Development and Pilot Testing of a Novel Electromechanical Device to Measure Wrist Rigidity in Parkinson’s Disease. Proceedings of the Annual International Conference of the IEEE Engineering in Medicine and Biology Society, EMBS.

[30] Xia R., Rymer W.Z. (2004). The Role of Shortening Reaction in Mediating Rigidity in Parkinson’s Disease. Exp. Brain Res..

[31] Caligiuri M.P. (1994). Portable Device for Quantifying Parkinsonian Wrist Rigidity. Mov. Disord..

[32] Teräväinen H., Tsui J.K.C., Mak E., Calne D.B. (1989). Optimal Indices for Testing Parkinsonian Rigidity. Can. J. Neurol. Sci./J. Can. Des. Sci. Neurol..

[33] Powell D., Muthumani A., Xia R. (2016). A Comparison of the Effects of Continuous versus Discontinuous Movement Patterns on Parkinsonian Rigidity and Reflex Responses to Passive Stretch and Shortening. J. Nat. Sci..

[34] Kwon Y., Park S.H., Kim J.W., Ho Y., Jeon H.M., Bang M.J., Koh S.B., Kim J.H., Eom G.M. (2014). Quantitative Evaluation of Parkinsonian Rigidity during Intra-Operative Deep Brain Stimulation. Bio-Medical Materials and Engineering, Proceedings of the 3rd International Conference on Biomedical Engineering and Biotechnology, Beijing, China, 25–28 September 2014.

[35] Little S., Joundi R.A., Tan H., Pogosyan A., Forrow B., Joint C., Green A.L., Aziz T.Z., Brown P. (2012). A Torque-Based Method Demonstrates Increased Rigidity in Parkinson’s Disease during Low-Frequency Stimulation. Exp. Brain Res..

[36] Meara R.J., Cody F.W.J. (1992). Relationship between Electromyographic Activity and Clinically Assessed Rigidity Studied at the Wrist Joint in Parkinson’s Disease. Brain.

[37] Rätsep T., Asser T. (2017). The Effect of Subthalamic Stimulation on Viscoelastic Stiffness of Skeletal Muscles in Patients with Parkinson’s Disease. Clin. Biomech..

[38] Rätsep T., Asser T. (2011). Changes in Viscoelastic Properties of Skeletal Muscles Induced by Subthalamic Stimulation in Patients with Parkinson’s Disease. Clin. Biomech..

[39] Marusiak J., Kisiel-Sajewicz K., Jaskólska A., Jaskólski A. (2010). Higher Muscle Passive Stiffness in Parkinson’s Disease Patients Than in Controls Measured by Myotonometry. Arch. Phys. Med. Rehabil..

[40] Costa P., Rosas M.J., Vaz R., Cunha J.P. (2015). Wrist Rigidity Assessment during Deep Brain Stimulation Surgery. Proceedings of the Annual International Conference of the IEEE Engineering in Medicine and Biology Society, EMBS.

[41] Peres L.B., Calil B.C., da Silva A.P.S.P.B., Dionísio V.C., Vieira M.F., de Oliveira Andrade A., Pereira A.A. (2021). Discrimination between Healthy and Patients with Parkinson’s Disease from Hand Resting Activity Using Inertial Measurement Unit. Biomed. Eng. Online.

[42] Lopes E.M., Sevilla A., Vilas-Boas M.D.C., Choupina H.M.P., Nunes D.P., Rosas M.J., Oliveira A., Massano J., Vaz R., Cunha J.P.S. (2019). IHandU: Towards the Validation of a Wrist Rigidity Estimation for Intraoperative DBS Electrode Position Optimization. Proceedings of the International IEEE/EMBS Conference on Neural Engineering, NER.

[43] Assis S., Costa P., Rosas M.J., Vaz R., Cunha J.P.S. (2016). An Adaptive Model Approach for Quantitative Wrist Rigidity Evaluation during Deep Brain Stimulation Surgery. Proceedings of the Annual International Conference of the IEEE Engineering in Medicine and Biology Society, EMBS.

[44] van den Noort J.C., Verhagen R., van Dijk K.J., Veltink P.H., Vos M.C.P.M., de Bie R.M.A., Bour L.J., Heida C.T. (2017). Quantification of Hand Motor Symptoms in Parkinson’s Disease: A Proof-of-Principle Study Using Inertial and Force Sensors. Ann. Biomed. Eng..

[45] Kim D.W., Hassett L.M., Nguy V., Allen N.E. (2019). A Comparison of Activity Monitor Data from Devices Worn on the Wrist and the Waist in People with Parkinson’s Disease. Mov. Disord. Clin. Pr..

[46] Mutlu N.O., Cenk Akbostanci M., Ari F., Karaarslan F.T. (2019). Development of Methods to Detect Movement Disorders in Parkinson’s Disease. Proceedings of the 3rd International Symposium on Multidisciplinary Studies and Innovative Technologies, ISMSIT 2019-Proceedings.

[47] Powell D., Hanson N., Joseph Threlkeld A., Fang X., Xia R. (2011). Enhancement of Parkinsonian Rigidity with Contralateral Hand Activation. Clin. Neurophysiol..

[48] Xia R., Sun J., Threlkeld A.J. (2009). Analysis of Interactive Effect of Stretch Reflex and Shortening Reaction on Rigidity in Parkinson’s Disease. Clin. Neurophysiol..

[49] Yardimci N., Cemeroglu O., Ozturk E., Gürlü G., Şahin E., Bozkurt S., Cengiz T., Karali G., Cakirbay H., Ilhan A. (2016). Median and Ulnar Neuropathy Assessment in Parkinson’s Disease Regarding Symptom Severity and Asymmetry. Park. Dis..

[50] Mendonça D.A., Jog M.S. (2008). Tasks of Attention Augment Rigidity in Mild Parkinson Disease. Can. J. Neurol. Sci..

[51] Meara R.J., Cody F.W.J. (1993). Stretch Reflexes of Individual Parkinsonian Patients Studied during Changes in Clinical Rigidity Following Medication. Electroencephalogr. Clin. Neurophysiol./Evoked Potentials.

[52] LR V.D., KE R. (1988). Interrater Reliability of a Clinical Scale of Rigidity. Phys. Ther..

[53] Bronte-Stewart H. (2021). Quantitative Digitography Solves the Remote Measurement Problem in Parkinson’s Disease. medRvix.

[54] Sepehri B., Esteki A., Ebrahimi-Takamjani E., Shahidi G.A., Khamseh F., Moinodin M. (2007). Quantification of Rigidity in Parkinson’s Disease. Ann. Biomed. Eng..

[55] Alves C.M., Rezende A.R., Marques I.A., Martins Naves E.L. (2021). SpES: A New Portable Device for Objective Assessment of Hypertonia in Clinical Practice. Comput. Biol. Med..

[56] Askari S., Zhang M., Won D.S. An EMG-Based System for Continuous Monitoring of Clinical Efficacy of Parkinson’s Disease Treatments. Proceedings of the 2010 Annual International Conference of the IEEE Engineering in Medicine and Biology Society, EMBC’10.

[57] Dural A., Atay M.B., Akbostanci C., Kucukdeveci A. (2009). Impairment, Disability, and Life Satisfaction in Parkinson’s Disease. Disabil. Rehabil..

[58] Lance J. (1980). Spasticity: Disorder of Motor Control.

[59] Aungst T.D., Clauson K.A., Misra S., Lewis T.L., Husain I. (2014). How to Identify, Assess and Utilise Mobile Medical Applications in Clinical Practice. Int. J. Clin. Pr..

[60] van den Bergh R., Bloem B.R., Meinders M.J., Evers L.J.W. (2021). The State of Telemedicine for Persons with Parkinson’s Disease. Curr. Opin. Neurol..

[61] Shalash A., Spindler M., Cubo E. (2021). Global Perspective on Telemedicine for Parkinson’s Disease. J. Park. Dis..

